# Effects of Systematic Categorization Training on Cognitive Performance in Healthy Older Adults and in Adults with Traumatic Brain Injury

**DOI:** 10.1155/2019/9785319

**Published:** 2019-07-29

**Authors:** Fofi Constantinidou

**Affiliations:** Department of Psychology and Center for Applied Neuroscience, University of Cyprus, Nicosia, Cyprus

## Abstract

This study investigated the effects of hierarchical cognitive training using the categorization program (CP), designed initially for adults with cognitive deficits associated with traumatic brain injury (TBI). Fifty-eight participants were included: a group of fifteen young adults with TBI (ages 18-48), another group of fifteen noninjured young adults (ages 18-50), and two groups of adults over 60 randomly assigned into the experimental group (*n* = 14) or the control group (*n* = 14). Following neuropsychological testing, the two young adult groups and the experimental older adult group received the CP training for 10-12 weeks. The CP training consisted of 8 levels targeting concept formation, object categorization, and decision-making abilities. Two CP tests (administered before and after the training) and three probe tasks (administered at specified intervals during the training) assessed skills relating to categorization. All treated groups showed significant improvement in their categorization performance, although younger participants (with or without TBI) demonstrated greater gains. Gains on the categorization measures were maintained by a subgroup of older adults up to four months posttraining. Implications of these findings in terms of adult cognitive learning and directions for future research on adult cognitive rehabilitation and cognitive stimulation programs are discussed.

## 1. Introduction

Several studies demonstrated that older adults benefit from cognitive training efforts that target specific processes. These findings create a paradigm shift because they suggest that the aging brain continues to be somewhat plastic and adaptable in old age. Improvement associated with training could be attributed to the development of a new skill or the facilitation of existing cognitive mechanisms and capacities [1, 2] that may be affected due to the aging process. Ultimately, cognitive training in aging could contribute to the increase of cognitive reserve, the brain's ability to withstand pathology [3].

Age-related cognitive changes are generally highly heterogeneous and are typically noted in tasks with high demands on speed of information processing and executive control (such as complex working memory tasks requiring manipulation of information), as compared to simple or automatic tasks (such as digit recall tasks) [3–6]. These changes in episodic memory can be measurable starting at age 30, and the rate of age-related decline in verbal episodic memory is normally mediated by working memory capacity [7]. The goal of cognitive training is to reduce the impact of the aging process on the cognitive system, [8] and in order to be successful, training programs should be based on sound theoretical models of cognition.

Contemporary cognitive theory organizes human cognition into a hierarchy of basic and complex processes or systems. Basic processes such as sensory perception, attention, and memory underlie more complex systems such as language, categorization, and executive functioning (for an extensive review, refer to Constantinidou and Thomas [9]). Deficits in categorization could interfere with the successful execution of daily activities because categorization skills are integral to memory and learning of new information and are essential processes for decision-making and successful problem-solving. Given the fundamental importance of categorization to all of intelligent behavior, it is surprising to observe the scarcity of investigation specific to the effects of aging on classification behavior. This is in contrast to other domains such as speed of processing and memory for which a substantial body of work can be found [10, 11]. The current study proposes to fill some of this gap in knowledge and is part of a systematic research program on the effects of categorization training on cognitive performance. Our previous research suggests that the CP is effective in young adults with TBI [12, 13]. The next paragraphs provide the theoretical framework for categorization-classification behavior relevant to this study.

The literature on categorization, especially that which is focused on visual object classification, divides into two largely nonoverlapping areas: those studies concerned with how we go about recognizing and categorizing ordinary objects in the world [14, 15] and those studies whose purpose is primarily to explain how novel situations or categories are acquired and later used to classify novel instances [16–18].

Regarding object perception, recognition, and categorization, semantic knowledge models support a bilateral temporal lobe hub which links object properties and conceptual understanding [19]. Furthermore, evidence in vision indicates the presence of a hierarchical recognition process that begins with early feature processing (such as orientation, motion, and color) and leads to the processing and representation of objects and object classes in the inferotemporal cortex [15, 20, 21]. Existing research suggests that both healthy aging and TBI result in changes in object classification [22, 23], indicating that treatment of object classification behavior should begin with feature identification in order to retrain the passive object recognition system [9].

For new category learning and classification of novel instance, empirical evidence suggests that people recruit one of two explicit systems (i.e., their processes and outputs are consciously available to the individual). The rule-based or rule-governed category system is the most important of the two. It involves the use of explicit verbalizable rules and hypothesis testing to determine category membership. This system relies heavily on executive functioning for its operation and engages frontal-subcortical networks. The other explicit system that requires significant episodic memory in that categorization is accomplished by the recall of previously experienced category members, or exemplars, that are similar to the present novel object [9, 17]. This latter system works by evaluating similarity, in terms of object or situation features, of to-be-categorized instances to those encountered previously [24], and its operation is sometimes labeled as procedural or nonexplicit, in the sense that one cannot easily describe the strategy used. Our knowledge of natural categories and common objects is likely to have been acquired through the use of the similarity-based system [20, 21, 25]. A long list of neuropsychological and experimental studies supports the above distinction [26–30]. The categorization program implemented in this study is organized hierarchically in order to address these two distinct areas of classification.

While the process of healthy aging is different from the pathology of TBI, healthy aging results in changes in categorization abilities, similar to TBI [22, 23]. Even though higher levels of cognitive reserve result in improved neurocognitive performance and moderate the effects of aging, age continues to be a robust predictor of neurocognitive functioning [3, 31].

Both TBI and age have been established as significant risk factors for the development of pathological aging resulting in dementia. TBI is now viewed as a chronic condition, and there is accumulating evidence suggesting that TBI and dementia of the Alzheimer's type share a common neuropathologic sequelae, such as chronic neuroinflammation (see review by Breunig et al. [32]), tauopathy [33], and the accumulation of beta amyloid (A*β*) [34], leading to significant neurocognitive impairment. Moderate-severe TBI sets off a neurodegenerative cascade as manifested by significant reductions in brain volume and lingering neurocognitive deficits [35] associated with longer time since injury placing the survivor at risk for dementia in middle/later life [36].

The current project responds to the challenge set by the NIH consensus report on preventing AD and cognitive decline [37]. The primary objective of this project was to investigate the utility of intense neurocognitive training in healthy older adults who experience neurocognitive changes associated with the normal aging process, using the categorization program (CP). A secondary objective was to compare the performance of older adults to younger healthy adults and to adults with cognitive deficits secondary to brain injury. Results from this study could guide future research investigating the effects of intense neurocognitive training on older adults with TBI and adults with mild cognitive impairment, the prodromal phase to dementia.

The CP is a rigorous systematic, hierarchical, eight-level program initially designed as a restorative cognitive rehabilitation program in adults with acquired brain injury. It addresses the aforementioned two distinct areas of human categorization, i.e., passive object recognition and new category learning. Initial research findings [12] and a subsequent randomized controlled trial [13] indicate that the CP is an effective therapy tool for adults with brain injury who exhibit categorization deficits. Constantinidou et al. [13] offered the following explanations regarding the active ingredients of the CP:

- The CP addresses both aspects of categorization, novel category learning, and categorization of established concepts or categories. It incorporates concrete visual stimuli and gradually progresses into abstract concepts through the use of repetition, cueing, and strategy building
- The CP was built using a very systematic hierarchical structure that corresponds to the neurodevelopmental order of categorization and classification process hierarchy
- Tasks gradually increase in difficulty and cognitive abstraction. CP tasks begin with basic feature identification and feature extraction (such as color, shape, and size) and progress to higher levels of concept formation and abstraction (such as rule-based decision-making) [38, 39]
- The program integrates cognitive processes such as executive skills, attention, organization, conceptual reasoning, linguistic flexibility, and explicit memory for the completion of the categorization tasks
- The redundancy and the repetition integrated in each level, along with the extensive cueing systems and errorless learning principles, provide support and organization for participants with more passive learning styles. The program provides a standardized approach to categorization training; yet, it incorporates mastery criteria for each level in order to account for individual differences. In conclusion, the CP targets cognitive domains such as complex working memory, information processing, and fluid intelligence that traditionally have been associated with age-related cognitive decline

The present study investigated categorization abilities and the effects of training on healthy older adults over 60 and in a group of young adults with moderate to severe TBI. In order to account for previous methodological flaws in adult training studies and determine the true effects of training, this study incorporated an older adult control group (who did not receive the training) and a young adult group who received training. The primary hypotheses were the following:

1. All participants who receive the CP training would demonstrate improvements in their categorization performance as measured by gains on the CP-dependent measures
2. Participants who receive the CP training would be able to generalize their knowledge to other categorization tasks not directly related to the CP, to a greater degree than participants who do not receive the CP training
3. Performance of older adults on formal neuropsychological measures measuring memory, executive function, perception, conceptual reasoning, and attention would correlate significantly with performance on categorization tests
4. Older adults who receive the CP training would be able to maintain their gains on categorization performance at one and four months posttraining

## 2. Materials and Methods

### 2.1. Participants

Fifty-eight participants were included in the study. One group consisted of young adults with TBI who received the CP training (*n* = 15), a second group of young healthy adults who also received the CP training (*n* = 15), a third group of healthy older adults who received the training (*n* = 14), and a fourth group of healthy older adults who did not receive the training (*n* = 14). Participants with TBI were recruited from brain injury rehabilitation centers collaborating in the project. The rest of the participants (groups 2-4) were volunteers from southwest Ohio areas. Recruitment of the TBI and noninjured groups was done in parallel and in a rolling admission process. All work was conducted in accordance with the Declaration of Helsinki (1964), and the project was approved by the Miami University Institutional Review Board for Human Subjects in Research. All study participants provided written informed consent prior to participation. Study participants were English-speaking adults and met the study's inclusion/exclusion criteria as follows.

### 2.2. Young Adults with TBI

The following are the inclusion/exclusion criteria for participants with TBI, which are consistent with the Constantinidou et al. [12] criteria.

#### 2.2.1. Inclusion Criteria

1. Adult males and females between 18 and 55 years of age
2. Primary diagnosis of moderate to severe CHI. The indication of an initial moderate to severe head injury was determined by the presence of three or more of the following severity indices: (a) initial Glasgow Coma Scale score less than 12, (b) abnormal initial computed tomography (CT) findings indicating acute central nervous system pathology, (c) length of impaired consciousness greater than 20 minutes as specified by the emergency records, (d) length of posttraumatic amnesia greater than 24 hours as specified in the acute hospital/emergency records, (e) length of acute hospital stay greater than 3 days, (f) positive neurological examination on hospital admission and discharge indicating focal sensory and motor neurological deficits or changes in the mental status attributed to brain injury, (g) medical complications secondary to the injury, and (h) head injury severity classification according to hospital records [40, 41]
3. Rancho Los Amigos Scale [42] Level VI or higher (which indicates appropriate, goal-oriented behavior and posttraumatic amnesia (PTA) resolution)
4. No aphasia present with the exception of mild to moderate word-finding problems due to cognitive deficits
5. Resolution of PTA as evidenced by a score of 76 or higher on the Galveston Orientation and Amnesia Test [43]
6. Enrollment in a residential comprehensive postacute rehabilitation program at the onset of the study
7. Participants were within 4 years of their injury

#### 2.2.2. Exclusion Criteria

1. Penetrating head injuries
2. Diagnosis of stroke at the time of injury
3. Premorbid central nervous system disorder or learning disability
4. Documentation of premorbid major depression or other significant psychiatric disorders as defined by the Diagnostic and Statistical Manual of Mental Disorders [DSM-IV] [44] that resulted in hospitalization and/or incapacity to work or perform activities of daily living
5. Current Beck Depression II [45] score of 25 or higher indicating the presence of depression that could interfere with performance on the protocol. This score is higher than the typical cut-off score of 15 because as previously described [12], TBI results increased symptomatology for reasons relevant to TBI and not because of clinical depression. A score of 25 or higher indicates clinical depression in this population
6. Active or current alcohol, drug, or other controlled substance abuse that interferes with participation
7. Deficits in auditory comprehension and moderate to severe word-finding problems, two standard deviations below the mean on the Boston Naming Test [46], which could interfere with the subject's ability to follow test or task instructions
8. English as a second language
9. Color blindness as measured by the Ishihara test for color blindness [47]

Seventy percent of the participants were injured in motor vehicle accidents, and another 30% were injured as a result of falls. All participants received comprehensive rehabilitation at the time of participation in the project.

### 2.3. Noninjured Adult Groups

#### 2.3.1. Inclusion Criteria

1. Adult males and females between 18 and 55 years of age for the young adult group
2. Adults over 60 for the older adult groups

#### 2.3.2. Exclusion Criteria

1. A medical history of a central nervous system trauma, disorder, or organic brain disease, learning disability, or language learning disability
2. Documentation of psychological or psychiatric disorder as defined by the DSM-IV [44] that resulted in hospitalization for major depression and/or incapacity to work
3. Current Beck Depression II [45] score of 15 or higher indicating the presence of depression that could interfere with performance on the protocol
4. Mini-Mental State Examination score of 25 or lower [48]
5. Active or current alcohol, drug, or other controlled substance abuse
6. Uncorrected visual or hearing deficits
7. Color blindness as measured by the Ishihara test for color blindness [47]

### 2.4. Group 1: Experimental Young Adult Group with TBI (*n* = 15)

Participants were residents of postacute rehabilitation centers and were enrolled in the project through a rolling admission process. They remained in the study for an average of 10-12 weeks which coincided with their length of stay at the rehabilitation centers. Participants in this group ranged in age from 18 to 48 years with a mean age of 28.13 (SD = 9.21). Education ranged from 12 to 17 years, with a mean of 13.67 (SD = 1.78).

### 2.5. Group 2: Young Adult Group (*n* = 15)

The ages of the participants ranged from 19 to 50 years with a mean age of 29.73 years (SD = 10.89). Education ranged from 12 to 18 years, with a mean of 14.16 (SD = 1.87).

### 2.6. Group 3: Experimental Older Adult Group (*n* = 14)

Participants ranged in age from 60 to 82 years with a mean age of 67.28 years (SD = 10.47). Education ranged from 12 to 19 years, with a mean of 13.9 (SD = 2.15). Their average MMSE score was 28.5 (SD = 1.55).

### 2.7. Group 4: Older Adult Control Group (*n* = 14)

Their ages ranged from 60 to 88 years with a mean age of 68.64 years (SD = 9.72). Education ranged from 10 to 18 years, with a mean of 13.92 (SD = 2.05). Their average MMSE score was 29.42 (SD = .85).

Healthy older adults who met the inclusion/exclusion criteria for the project were randomly assigned to either the experimental or the control group. The two older adult groups did not differ significantly in age or education level, *t*(26) = .355, *p* = .725 and *t*(26) = .043, *p* = .966. There was no difference in their gross cognitive ability as measured by the MMSE *t*(26) = 1.958, *p* = .061. Finally, there was no significant difference between the four groups on the years of education, *F*(3, 54) = .154, *p* = .927.

### 2.8. Procedures

Participants were administered a neuropsychological assessment at the beginning of their participation in the project. Following the neuropsychological testing, participants were administered two categorization tests designed for this project and the first probe task (see sections 2.11 and 2.12 for a description of the tests and probe tasks). All participants remained in the study for 10-12 weeks. Participants who received the CP training (groups 1, 2, and 3) participated in individualized hourly sessions for a total of 2-4 hours of training per week until they completed the CP protocol. On average, participants required 27 hours to complete the CP training.  Table 1 displays the experimental design.

### 2.9. Experimental Items

The experimental items are consistent with the materials described previously [12, 13], and the description partly reproduces their wording:

### 2.10. The Categorization Program (CP)

The CP was based on theories of implicit and explicit categorization systems. Therefore, tasks were grouped into two major parts: (1) recognition and categorization of everyday objects and (2) new category learning [12]. Principles of learning, concept formation, and rehabilitation were incorporated in order to develop the hierarchical tasks [49–52].  Table 2 presents the 8 levels of the CP.

#### 2.10.1. Part A: Object Categorization Tasks

This part consists of 5 different levels. The tasks begin with teaching perceptual features in order to describe objects or living things and move to higher levels of cognition including analyses, synthesis, linguistic flexibility, and abstract reasoning.

#### 2.10.2. Part B: New Category Learning Tasks

The new category learning tasks consist of three levels. Under each level, there are 5 steps that increasingly demand a higher level of rule-governed responses. Errorless learning principles and cueing hierarchies are applied under each step.

The CP-dependent measures were developed to measure the effectiveness of the CP program. These were the CP Test 1, CP Test 2, and probe tasks 1, 2, and 3.

### 2.11. CP Tests 1 and 2

CP Test 1 relates to the categorization of common objects (Part A of the CP). Participants were required to describe pictures of objects and identify core attributes such as their primary function and alternate uses of the object. These objects were not part of the CP training. There were a total of 10 objects; five have high frequency and five have low frequency in occurrence [53]. The number correctly obtained from 120 possible was recorded for each subject. Cronbach's alpha for the 10 items is .90.

The second test relates to the new category learning portion (Part B of the CP). Participants were required to follow a logical rule in categorizing objects. These objects were not part of the CP. There were a total of 5 rules with increased complexity. For instance, the first rule asks the subject to “put all red items in the basket.” The last rule is more complex and requires that participants to “put all things that are blue but not used for coffee in the basket.” Both informal tests were administered at the beginning and at the end of the study. The number correctly obtained from 36 possible was recorded for each subject. Cronbach's alpha for CP Test 2 was .69.

### 2.12. CP Probe Tasks

The probe tasks were designed to assess how participants generalize information learned on the CP to other tasks not directly related to the CP training tasks. Participants were presented with an array of 10 objects and were required to categorize objects based on a self-generated rule. Following that, they were asked to categorize the same objects twice, each time using a different self-generated rule.

The probe tasks were administered at 3 different times during the study. The first probe task was administered prior to the CP training, the second after Level 2 (Part A), and the third after Level 5 (Part A). For the treated control group participants, the first probe was administered prior to the onset of therapy, the second after 5 weeks of treatment, and the third after 8 weeks of treatment. Each of the probe task uses a set of 10 different objects, equal in familiarity and frequency of occurrence. The three different sets of probe tasks are of similar difficulty, and analyses of variance yielded no statistical difference in performance (*p* > .05) between the three different sets of items. The order of administration was counterbalanced to avoid order effects. Participants obtained one point for each object they sorted correctly by their self-generated rule. The subject was asked to sort the items three times under each probe task, each time using a different rule. Hence, each probe trial was worth 10 points with a total of 30 points for each probe task.

### 2.13. CP Stimuli and Scoring System

The CP protocol was provided in a manualized format in order to ensure consistency. The CP packet included the administration manual with cueing instructions, the CP stimuli (objects, photos, written words, etc.), and score sheets.

### 2.14. Neuropsychological Tests

The following tests were administered at pre- and posttesting.

- Wechsler Abbreviated Scale of Intelligence (WASI) [54]
- Mini-Mental State Examination [55]
- Rey Complex Figure Test [56]
- Trail Making Tests A and B [57]
- Digit Span Forward and Backwards and Visual Span Forward and Backwards—*Wechsler Memory Scale-III* (WMS-III) [58]
- California Verbal Learning Test-II [59]
- Wisconsin Card Sorting Test [60]
- The Booklet Category Test [61]
- Symbol Digit Modalities Test [62]
- Control Oral Word Association [63]
- The Picture Recognition, Spatial Relations, Analysis and Synthesis, Concept Formation, Decision Speed, and Verbal Comprehension Subtests from the Woodcock-Johnson III (WJIII, Tests of Cognitive Abilities) [64]

### 2.15. Data Scoring and Analyses

Data were included in the analyses to the fullest extent possible. The primary statistical design was a multivariate mixed model design followed by preplanned univariate comparisons. The alpha level was set at .05.

## 3. Results

Participants who received the CP protocol required about 27 hours of treatment spread over 10-12 weeks. The participants in the control group did not receive any cognitive treatment other than information regarding memory strategies and a list of suggested activities for cognitive stimulation.

### 3.1. Performance on CP Measures

CP measures consisted of the CP Test 1 (pre and post), CP Test 2 (pre and post), and the three probe tasks (Probe 1, Probe 2, and Probe 3).

#### 3.1.1. CP Test 1

The first CP test assessed the ability to categorize common objects. In order to determine the effects of the CP training on categorizing familiar objects, a mixed model analyses of variance (*a* = .05) compared the pre- and postperformance of the four groups on the CP Test 1 with time (pre/post) as the within-subjects factor and group as the between-subjects factor. The analyses revealed a significant time effect (*F*(1, 54) = 146.14, *p* = .0001, *ηρ*^2^ = .730, power = 1.000), group by time interaction (*F*(3, 54) = 24.72, *p* = .0001, *ηρ*^2^ = .579, power = 1.0), and an overall group effect (*F*(3, 54) = 11.411, *p* = .0001, *ηρ*^2^ = .388, power = .999).

Planned lower-order ANOVAs indicated that there was an overall group difference on the CP Test 1 baseline performance (*F*(3, 57) = 6.026, *p* = .001). However, there was no difference at the baseline among the three healthy groups on CP Test 1 accuracy (*F*(2, 42) = .187, *p* = .831). Post hoc pairwise Bonferroni comparisons indicated that the baseline difference at CP Test 1 was due to the lower performance of the young group with TBI. Participants with TBI performed significantly lower on CP Test 1 at the baseline as compared to the healthy young adults and the two older adult groups.

**Figure 1:**
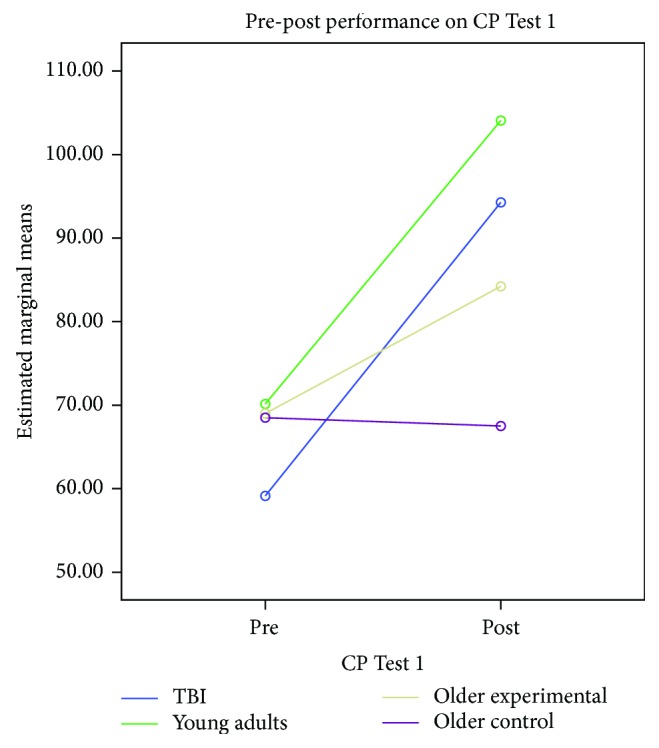
CP Test 1 interaction and pre-post group effects.

At Time 2, there was a significant group main effect (*F*(3, 54) = 19.785, *p* = .0001). Post hoc pairwise Bonferroni comparisons revealed no difference between the TBI and the healthy young adult or the TBI and the older experimental group at Time 2. However, healthy young adults performed significantly better than their older counterparts who received the CP training. The older untreated group had the lowest performance among all groups at Time 2, and their performance was significantly lower than that of participants in the other groups (e.g., TBI, young adult, and older experimental groups). While all *treated* groups demonstrated significant growth from Time 1 to Time 2, the young adults (TBI and healthy young adults) demonstrated the most significant growth as compared to the older treated group, *t*(27) = 3.585, *p* = .001 and *t*(27) = 3.576, *p* = .001. The degree of improvement on CP Test 1 (i.e., difference score between pre-post performance) between the two young adult groups was similar (*t*(28) = .209, *p* = .836).  Figure 1 displays time by group interaction depicting the change in performance from Time 1 to Time 2 on the CP Test 1 tasks.  Table 3 presents the performance of each group on the CP-dependent measures.

#### 3.1.2. CP Test 2

The second CP test assessed the ability to implement logical rules to categorize objects consistent with theories of category learning. In order to determine the effects of the CP training on implementing logical rules, a mixed model analyses of variance (*a* = .05) compared the pre- and postperformance of the four groups on the CP Test 2 with time (pre/post) as the within-subjects factor and group as the between-subjects factor. There was a time main effect (*F*(1, 53) = 13.521, *p* = .001, *ηρ*^2^ = .203, power = .950) and a group main effect (*F*(3, 53) = 4.830, *p* = .005, *ηρ*^2^ = .215, *p* = .883). The group by time interaction was not significant (*F*(3, 53) = .756, *p* = .524, *ηρ*^2^ = .041, power = .201). Results indicate that the patterns of performance were similar among the groups. Planned lower-order ANOVAs indicated that there was a difference at the baseline between the four groups (*F*(3, 53) = 2.964, *p* = .040). The only significant difference was between the noninjured young adults and the two older adult groups (*F*(2, 41) = 4.086, *p* = .024). There were no other significant group differences at the baseline.

**Figure 2:**
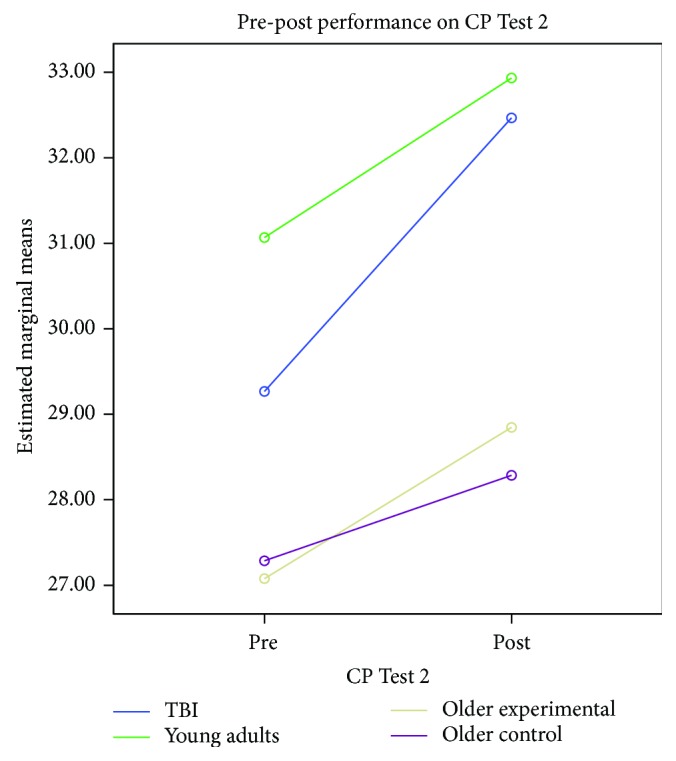
CP Test 2 pre-post group effects.

At Time 2, there was a significant group simple main effect (*F*(3, 54) = 5.223, *p* = .003). A priori pairwise comparisons (*α* = .01) revealed significant differences between the young TBI group and the treated older adult group (*t*(27) = 2.814, *p* = .009) and between the healthy young adult group and the treated older adult group and untreated older adult groups (*t*(27) = 2.839, *p* = .009 and *t*(27) = 2.78, *p* = .010, respectively).  Figure 2 displays group performance on the CP Test 2.

### 3.2. Probe Tasks

The probe tasks were designed to assess the participants' ability to implement skills learned during the CP training and categorize new objects. The three probe tasks were administered at three different intervals: before the CP training, after Level 2 (at 5 weeks after the onset of treatment), and after Level 5 (at 8 weeks after the onset of treatment). Participants in the control group received the probes before the onset of the study (i.e., baseline), at 5 weeks, and at 8 weeks.

A mixed model analysis of variance was performed with probe as the within-subjects factor and groups as the between-subjects factor. There was a significant probe effect (*F*(2, 51) = 8.536, *p* = .001, *ηρ*^2^ = .251, power = .958), group by probe interaction (*F*(6, 104) = 2.422, *p* = .032, *ηρ*^2^ = .127, power = .801), and a significant group main effect (*F*(3, 52) = 3.238, *p* = .029, *ηρ*^2^ = .158, power = .715).

Pairwise analyses (mean difference estimations, *a* = .01) indicate that there was a significant improvement between the first and second probe administrations (*p* = .001). There was also a significant difference between the first and third probes (*p* = .0001). However, there was no significant difference between the second and third probes (*p* = .291).

**Figure 3:**
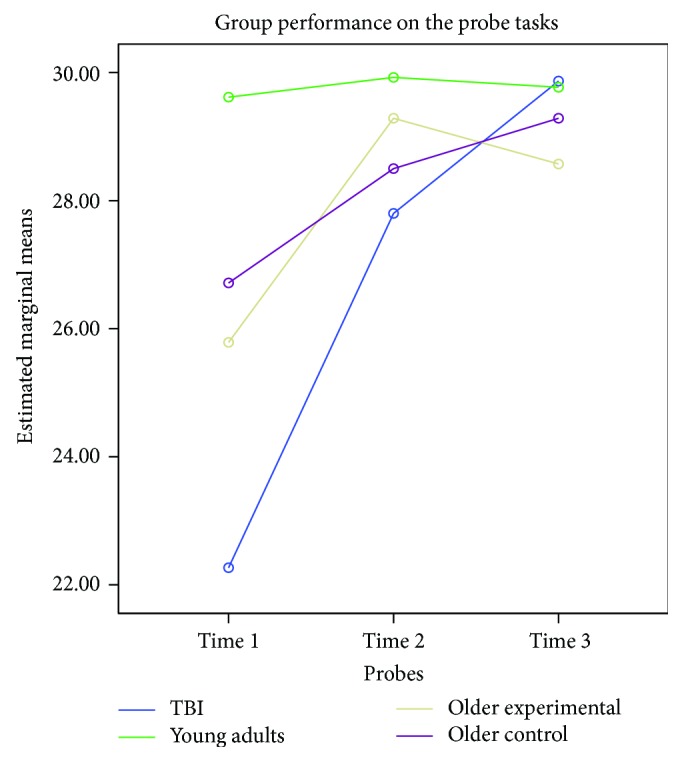
Probe effects per group across time.

The groups demonstrated different patterns on performance on the probe tasks. The greatest gain in performance was obtained by the young adult group with TBI (*F*(2, 13) = 7.734, *p* = .006, *ηρ*^2^ = .543, power = .887). The performance of young adults with TBI and older adults during Probe 1 was more variable (compared to younger adults). However, their performance during Probes 2 and 3 became more uniformed. The performance of the young adult group was fairly stable across the three probes, probably due to a ceiling effect.  Figure 3 displays group performance on the probe task across time.

### 3.3. Follow-Up Effects of CP Training

Out of the 14 older participants who received the CP training, 9 participants were able to complete follow-up assessments at one month and at 4 months after study completion in order to determine possible long-term effects of the CP training. The remaining five dropped out because they missed one of the two follow-up sessions due to illness, planned surgeries, death in the family, and travel. A repeated measures ANOVA (*a* = .05) on the CP Test 1 scores obtained during the four different administration times (pretest, posttest, follow-up Time 1, and follow-up Time 2) revealed significant differences in performance (*F*(3, 24) = 11.69, *p* = .001, *ηρ*^2^ = .593, power = .0001). Preplanned Helmert contrasts indicated a significant difference in performance between the pretest score (*x* = 72.11, SD = 4.85) and the rest of the posttest scores (*F*(1, 8) = 16.94, *p* = .002, *ηρ*^2^ = .679, power = .948) (posttest average score = 87.11, SD = 12.91; one − month average score = 95.56, SD = 17.16; and four − month average score = 92.33, SD = 16.04). There was no significant difference between the three posttest scores on CP 1 (*F*(2, 7) = 3.832, *p* = .075, *ηρ*^2^ = .523, power = .499).

Similar to the CP Test 1, a repeated measures ANOVA (*a* = .05) on the CP Test 2 scores obtained over four different administration times (pretest, posttest, follow-up Time 1, and follow-up Time 2) revealed a significant overall effect (*F*(3, 24) = 4.44, *p* = .013, *d* = .357, power = .816). There was no significant difference between the three posttest scores on CP Test 2 (*F*(2, 7) = 1.581, *p* = .271, *ηρ*^2^ = .311, power = .231) (posttest average score = 28.38, SD = 5.13; one − month average score = 30.33, SD = 3.96; and four − month average score = 30.33, SD = 5.26). In summary, the small group of participants who received the follow-up testing maintained their gains on CP Tests 1 and 2 after the training up to 4 months after the study completion.

### 3.4. Pretest Neuropsychological Measures and Performance on the CP Tests

One objective of the present study was to determine the relationship between neuropsychological performance and the CP-dependent measures for the older adults. Scores from the full set of baseline neuropsychological assessments were combined into a set of five composite scores representing the conceptually motivated constructs [13, 65] of Memory Processing, Executive Functioning, Perceptual/Visual Processing, Conceptual Reasoning, and Organization/Attention using a method advocated by Cahn and colleagues [66] and Cohen and colleagues [67]. Each measure was converted into a *z*-score. The resulting *z*-scores for the measures within each construct were then averaged to derive a score for the constructed measure. The two Executive Function measures (Wisconsin Card Sorting Test: Trials to First Category, Total Number of Categories, and Failure to Maintain Set), which were not significantly correlated with the Executive Function composite variable, were removed from the composite, and the Executive Function composite was recomputed from the remaining measures in that set.  Table 4 presents the group means on the pre- and postneuropsychological measures. Values under means are standard deviations; Table 5 displays the correlations between individual measures and their composite scores.

The correlation between the pretest scores of CP Test 1 and CP Test 2 was weak and nonsignificant (*r* = .182, *p* = .087) because the two dependent measures assessed different categorization constructs. The left columns of Table 6 contain the Pearson product-moment correlations between the composite scores and the categorization pretest measures (CP Test 1, CP Test 2, and Probe 1) for both groups of older adults. At Time 1 (pretest), CP Test 1 performance correlated significantly with all five composite indices; CP Test 2 correlated significantly with all indices except the Organization/Attention Composite. Probe 1 correlated with all the composite scores except the Perception/Visual Processing composite.

Finally, in order to examine whether neuropsychological measures could predict who benefited most from the categorization program, the *z*-score composite measures were correlated with the three difference scores (CP Test 1 posttest minus pretest, CP Test 2 posttest minus pretest, and Probe 3 minus Probe 1) using the data from the older adults who received the CP training. Additionally, partial correlations (using the pretest score as a covariate) between the composite scores and the categorization posttest measures (CP Test 1, CP Test 2, Probe 2, and Probe 3) were conducted. As can be seen in Table 6, the Perceptual/Visual, Conceptual/Reasoning, and Global Cognitive were the most useful composites in predicting improvement in performance on the CP-dependent measures.

## 4. Discussion

The current study is part of a systematic research program exploring the effects of a hierarchical cognitive training program in adult rehabilitation. The primary objective of the present study was to determine the utility of such training in healthy older adults and compare their performance to healthy young adults and to young adults with known neurocognitive deficits resulting from TBI. The results support the notion that older adults similar to adults with TBI can benefit from cognitive activities that enhance organization and conceptual knowledge. Overall, the three groups of participants who received the CP training demonstrated improvement in their categorization performance. Categorization in the project was measured directly by the two CP tests developed specifically for this training program. Participants who received the CP training showed improvement in their ability to categorize common objects as measured by their performance on CP Test 1. During CP Test 1, participants were required to describe objects effectively and to generate creative uses that could improve functional problem-solving abilities. Participants in the experimental groups demonstrated significant gains in describing common objects and in creative uses of objects. In comparison, participants in the control group did not demonstrate significant gains in this area. Greater gains however were noted in the young adults with TBI and young healthy adults, in comparison to the older experiment adult group.

CP Test 2 measures the ability to categorize based on predetermined rules. During this test, participants were provided the rule and asked to classify objects based on the rule. This type of task is considered passive in nature because participants were not required to delineate the rule themselves. In contrast, during the actual training tasks of the CP, participants were required to delineate the rule based on feedback they received from the clinician. While young participants (both with TBI and noninjured young adults) demonstrated significant improvement on the CP Test 2, older adults who received the training did not demonstrate significant gains. Therefore, it appears that older adults demonstrate greater gains in the classification of objects as compared to rule-based learning.

Older adults were followed up to four months upon completion of their training. This was not possible for the TBI group since completion of the CP training also coincided with discharge from the rehabilitation facility. Findings indicated that older adults who received the training were able to maintain their gains at one and four months posttraining. Future studies need to explore the posttraining effects systematically. This information can guide future research on the CP and on potential benefits of “tune-up” sessions at specified intervals.

One of the primary challenges faced by cognitive rehabilitation programs centers on the realization that knowledge acquired during training may not transfer into other novel (untrained) tasks. Transfer success may be influenced by a number of factors stemming from the nature of the tasks, types of stimuli, and cognitive distance between the trained task and the untrained task as well as unfamiliarity with the untrained task itself. This challenge in transferring skills from domain-specific to more general can limit the practical impact of cognitive training programs as well as the motivation of participants to continue their training. An additional variable that contributes to the aforementioned challenge might be the advanced age of some older participants (i.e., over 75), which in turn could create difficulties in learning, speed of information processing, mental flexibility, and reduction in strategy use [8].

Hence, another important objective of the present study was to determine whether the CP training facilitates the ability to generalize categorization skills to novel tasks as measured by the probes. The current results indicated that our older adult group, similarly to adults with TBI, demonstrated gains in the probe performance. However, the gains of the young TBI group were greater than that of the older adult group. This might be due to the fact that the injured TBI group had a greater room for improvement on this task. Future studies may want to increase the complexity of the probe tasks in order to ensure that they provide opportunity for improvement in performance as a result of training.

### 4.1. Relationship between the CP Measures and Formal Neuropsychological Tests

Given the focus of CP Test 1 on object recognition and categorization, it is understandable that the skills this test measures overlap significantly with those assessed by the neuropsychological tests comprising the Perception Composite score and the Conceptual/Reasoning composite. On the other hand, rule-governed categorization has been consistently related to executive functioning, attention, and reasoning processes using a variety of methodologies [27, 29]. The present findings are largely consistent with this view as can be seen by the fact that composite measures having to do with executive function, attention, and reasoning correlate with performance on CP Test 2 during the pretest and posttest administrations. Probe task improvement is largely related to conceptual/reasoning and perceptual/visual measures, although this task may be affected by a ceiling effect. Finally, the Memory Composite and the Global Cognitive composite scores relate to all CP-dependent measures. The present findings with older adults are consistent with our previous work with the CP and TBI [13].

Future research should continue to explore the interaction between task properties and learner capacity. Evidence suggests [68] that individuals with high working memory capacities (as measured, for instance, by the digit span task) will learn rule-based problems faster than they can learn problems requiring a nonexplicit, similarity reasoning strategy. Alternatively, individuals with lower working memory capacity learn the nonrule-governed category structures faster than they learn those defined by rules. In the present study, baseline performance on the CP-dependent measures was highly correlated with the memory composite scores. This suggests that it may be profitable, in the designing of neurocognitive programs like the CP, to understand the relationship between individual neuropsychological characteristics and performance in categorization retraining tasks. The present study implemented composite scores in order to create theoretically meaningful constructs and also as a strategy to reduce the number of comparisons. Due to the preliminary nature of the findings and the small sample size, further corrections for multiple comparisons were not implemented. This limitation should be taken into consideration when interpreting the above findings.

### 4.2. Conclusions, Clinical Implications, Limitations, and Future Research

The present results contribute to the growing body of literature supporting cognitive training in healthy older adults. Improvements noted by cognitively healthy older adults and by younger participants with TBI, are consistent with our previous work implementing the CP with younger adults who sustained neuropsychological deficits secondary to closed head injury [12, 13]. However, gains demonstrated by older adults are not as dramatic as those demonstrated by their younger counterparts. Future research needs to determine the utility of the CP in treating older patients with TBI during rehabilitation and in the chronic phase post injury. This is in light of recent evidence on the neurodegenerative effects of chronic TBI on the brain structure and function [35, 69].

The Task Force on Promotion and Dissemination of Psychological Procedures and the World Health Organization define efficacy research as the examination of an intervention's effect under highly controlled experimental conditions [50, 70]. The current results suggest that the CP training is efficacious in enhancing certain aspects of categorization performance. The CP training implemented a standardized, manualized protocol and followed the necessary procedures in order to adhere to the standards and rigor of experimental research. However, given the small sample sizes in the present study, the results should be interpreted with caution and could not be generalized to the larger population of TBI and older adults. Future research should incorporate larger sample sizes to reduplicate these findings and also incorporate quality of life outcome measures in order to determine the potential generalizability of cognitive training in other aspects of daily activities.

One of the strengths of the current project was the careful selection of participants in order to create a homogeneous sample of subjects, improve internal validity, and reduce variability in performance. In addition to determining the utility of the CP training in older adults with TBI, future research should include adults with mild cognitive decline in order to determine the potential utility of this type of training in adults who may be at risk for developing dementia.

Present findings with a small subgroup of older adults demonstrate that the positive effects of CP training last for up to 4 months post treatment. This finding can guide future research on determining the timing and potential benefit of periodic booster sessions. Additionally, the link between CP training and improving cognitive reserve in older adults may be a fruitful line of research. Furthermore, future studies exploring the long-term effects of treatment should incorporate older adults who do not receive the training in addition to both younger and older adults with TBI.

Biological aging does not seem to be a uniformed process; therefore, larger studies (with neurologically healthy and neurologically compromised groups of participants) will afford the necessary statistical power to identify subgroups of older adults and delineate who benefits the most from this type of training. The identification of individuals with specific neuropsychological and genetic (i.e., ApoE allele) profiles, determination of mitigating factors, and the development of predictive models indicating who would demonstrate the greatest gains would be an important contribution of larger scale studies.

In closing, the present results provide additional evidence supporting the continued investigation of the CP training in adult neurorehabilitation. Future larger scale clinical trials would provide stronger evidence for the CP utility. The long-term effects of CP training on categorization performance and the use of CP training in older participants who are at risk for dementia and in older adults with TBI would be a fruitful line of investigation.

## Figures and Tables

**Table 1:** Experimental design.

			Part A							Part B	
	Pretests	Probe 1	Perceptual feature training Level 1	Similarities and differences Level 2	Probe 2	Functional categorization Level 3	Analogies Level 4	Abstract concepts Level 5	Probe 3	Levels 1-3	Posttests
Group 1: young adults with TBI (treated)	✓	✓	✓	✓	✓	✓	✓	✓	✓	✓	✓
Group 2: young uninjured adults (treated)	✓	✓	✓	✓	✓	✓	✓	✓	✓	✓	✓
Group 3: older adults (treated)	✓	✓	✓	✓	✓	✓	✓	✓	✓	✓	✓
Group 4: older adults (untreated)	✓	✓			✓				✓	✓	✓

Note: pre/posttests include the neuropsychological assessment and the 2 tests of the categorization program. Probe 2 was inserted at about 5 weeks after the onset of treatment and probe 3 at 8 weeks for the control group.

**Table 2:** The eight levels of the categorization program (adapted from Constantinidou et al. [38] and Constantinidou et al. [12]).

Part A: object categorization tasks	This part consists of 5 different levels. The tasks begin with teaching perceptual features in order to describe objects or living things and move to higher levels of abstraction.
Level 1: perceptual feature training and application	The purpose of this section is to train perceptual feature identification thereby building a framework for cognitive structures. The retraining of basic categorization abilities will build the foundation for more abstract functions and will facilitate communication during word-finding difficulties. The patient will learn eight perceptual features and then consistently apply all the features to describe common objects. Objects are presented via a range of stimulus types including real objects, color photos, line drawings, written words, and spoken words.
Level 2: similarities and differences	The purpose of this level is to apply the eight perceptual features trained in Level 1 to compare objects. Identification of similarities and differences between two objects of the same and of different categories using the eight perceptual features is utilized in order to train conceptual thinking. The process of applying the trained perceptual features is the next layer of the continuum of concrete to abstract functional abilities. Stimulus types include colored photos, written words, and spoken words.
Level 3: functional categorization	The purpose of this task is to identify functional categories and maintain the delineations within that category. There are two specific foci in this level which require the consideration of the features of the objects trained and applied in Levels 1 and 2: the application of retrieval strategies to generate novel items that belong in a given category and the mental flexibility required to generate alternate uses for the objects in a given category. This task enhances functional problem-solving and mental flexibility.
Level 4: analogies	The purpose of this level is to apply both the categorization abilities trained in Levels 1-3 and inductive reasoning skills in order to identify and match the concepts represented in analogies. The analogies progress from concrete to abstract in order to train word abstraction. Stimulus materials include multiple choice responses for each analogy that will aid in the training process of word abstraction as needed.
Level 5: abstract word categorization	This level further develops concept formation and abstract conceptual thinking. The goal is to identify similarities and differences in abstract verbal concepts. The generation of similar word pairs using synonyms that represent the relationship between the words is incorporated to enhance cognitive and linguistic flexibility.
Part B: new category learning tasks	Under each level of the new category learning, there are 5 steps that increasingly demand a higher level of rule-governed responses. Errorless learning principles and cueing hierarchies are applied under each step.
Level 1: progressive rule learning 1	The stimuli for Level 1 vary along two dimensions: shape and color. The nine stimuli include squares, circles, and triangles that are red, white, and black. Each stimulus is presented individually, and a formulation of the rule that classifies each stimulus into either Category A or Category B follows.
Level 2: progressive rule learning 2	The stimuli for Level 2 of Part B are gauges that include two dials that must be interpreted as a single unit. This level forces generalization into a real world situation by simulating the reading of gauges at a power plant. The determination of operational or not operational for each stimulus is utilized, and the cumulative interpretation of each judgment leads to the formulation of the rule that classifies the stimuli for each of the five conditions.
Level 3: progressive rule learning 3	The final explicit rule task contains the same underlying structure as the earlier two levels; however, this time, a judgment is made using stimuli constructed from dimensions of language. This further abstracts the rule formulation and forces generalization of training to a real world situation. The stimuli in this task consist of a summary of three laboratory tests (lung capacity, heart fluid, and bone marrow count) and their orthogonal combination with two measurement adjectives (low and high).

**Table 3:** Mean (standard deviations) for CP Tests 1 and 2 and the probe tasks.

	CP Test 1		CP Test 2		Probes		
	Pre	Post	Pre	Post	Probe 1	Probe 2	Probe 3
Group 1: young adults with TBI (treated) (*n* = 15)	59.13 (9.47)	94.26 (17.91)	29.26 (3.82)	32.46 (2.29)	22.26 (7.59)	27.8 (4.07)	29.86 (.51)
Group 2: young uninjured adults (treated) (*n* = 15)	70.13 (8.77)	104.21 (14.75)	31.06 (3.39)	32.93 (3.43)	29.64 (.84)	29.33 (.25)	29.78 (.57)
Group 3: older adults (treated) (*n* = 14)	69.0 (7.93)	84.21 (12.02)	27.07 (4.40)	28.35 (5.13)	25.78 (6.11)	29.28 (2.67)	28.57 (3.63)
Group 4: older adults untreated (*n* = 14)	68.50 (4.76)	67.50 (3.67)	27.28 (4.81)	28.28 (5.41)	26.71 (6.0)	28.5 (2.67)	29.28 (2.67)

Note: the maximum possible score on CP Test 1 is 120, and on CP Test 2 is 36. The total number of possible points for each probe task is 30.

**Table 4:** Performance on neuropsychological measures.

	Group 1: young adults with TBI (treated) *n* = 15		Group 2: young uninjured adults (treated) *n* = 15		Group 3: older adults (treated) *n* = 14		Group 4: older adults (untreated) *n* = 14	
	Pre	Post	Pre	Post	Pre	Post	Pre	Post
*California Verbal Learning Test-R (CVLT-R)*								
Total trials 1-5	51.0	55.5	58.93	65.80	47.14	49.64	45.43	49.50
	14.06	11.32	7.85	6.07	9.77	10.49	9.87	9.02
Short delay	8.27	11.1	12.33	14.80	8.92	10.54	9.57	11.00
	4.81	5.13	2.09	2.00	3.66	4.39	3.00	3.59
Long delay	9.27	11.6	14.93	13.60	10.92	9.00	11.14	9.50
	4.45	3.89	1.53	1.72	3.50	3.94	3.44	3.16
*Rey Complex Figure (RCF)*								
Copy	29.61	31.09	34.90	34.53	29.82	30.36	30.00	32.00
	7.38	4.67	1.56	2.36	6.47	6.72	6.10	3.68
Immediate	16.34	22.77	21.64	28.71	13.50	15.93	10.32	16.64
	6.83	5.47	4.57	5.39	8.20	8.27	5.03	5.92
Delayed	17.34	22.9	21.77	27.63	12.04	15.39	10.50	16.0
	6.33	5.26	4.74	6.44	5.96	8.81	5.88	5.72
*Wechsler Memory Scale-III (WMS-III)*								
Longest Digit Span Forward	7.5	8.6	6.53	6.93	6.78	6.57	6.57	6.36
	2.61	2.36	1.41	1.33	1.31	1.16	1.45	.84
Spatial Span Forward	8.28	9.54	8.93	9.27	7.79	8.00	7.50	7.78
	1.58	1.29	2.25	2.09	2.29	1.88	1.83	1.19
Spatial Span Backward	7.85	8.72	8.27	8.27	7.50	7.14	6.64	6.21
	1.65	1.67	2.02	1.79	1.45	1.83	1.39	1.05
*Wisconsin Card Sorting Test (WCST)*								
Number of Categories Completed	5.78	6.0	5.20	5.73	4.93	4.43	4.57	5.29
	.42	.0	1.57	.80	1.82	2.24	1.70	1.44
Trials to Complete First Category	13.21	11.36	14.67	13.27	17.43	12	16.28	16.28
	5.72	1.56	6.42	4.58	22.62	4.45	9.86	13.76
Failure to Maintain Set	.57	.27	.47	.27	1.14	1.14	.71	1.07
	.64	.46	.64	.59	1.29	2.03	1.07	1.64
*Booklet Category Test*								
Total errors	40.0	27.4	33.53	18.20	70.07	54.79	65.07	56.64
	20.58	14.02	25.90	17.04	21.54	24.15	28.96	31.04
*Symbol Digits Modality Test*	40.25	47.75	58.20	65.47	44.50	48.07	44.00	47.00
	10.11	10.71	9.51	13.17	15.00	11.59	9.37	11.71
*Controlled Oral Word Association Test(COWAT)*	33.1	41.7	42.73	49.47	39.29	39.78	41.93	41.86
	8.67	13.04	9.50	9.34	8.30	10.02	17.23	17.06
*Trail Making Test A (in seconds)*	36.16	38.98	25.67	23.10	34.61	35.36	39.93	36.86
	11.37	44.45	6.98	6.60	11.24	14.78	14.61	13.70
*Trail Making Test B (in seconds)*	79.5	90.54	57.45	55.80	79.43	74.57	74.21	86.57
	45.5	85.4	23.20	24.90	26.42	26.37	25.82	43.95
*Woodcock-Johnson III (WJIII-13)*								
Picture Recognition	48.42	51.42	53.33	54.53	51.86	50.43	51.21	51.86
	3.36	3.75	3.02	2.75	4.15	8.91	3.98	3.35
*WJIII-3*								
Spatial Relations	70.21	73.64	74.07	74.87	68.50	68.64	68.29	69.50
	5.1	5.41	6.28	5.45	6.34	9.64	7.18	6.46
*WJIII-15*								
Analysis/Synthesis	27.78	28.71	28.67	28.93	23.37	25.00	23.71	23.36
	2.42	2.16	4.35	4.68	6.06	5.72	4.07	7.24
*WJIII-5*								
Concept Formation	28.35	34.85	36.57	37.21	28.64	29.93	28.21	28.71
	5.3	4.18	2.93	5.29	7.80	6.76	6.04	7.50
*WJIII-16*								
Decision Speed	26.0	31.78	36.46	38.15	32.07	34.14	30.14	32.29
	8.35	7.29	5.59	3.29	5.81	6.02	6.19	6.13
*WJIII-1*								
Verbal Comprehension	52.92	56.14	59.57	61.50	56.43	56.78	58.28	57.93
	5.99	5.9	4.62	4.83	5.65	6.18	5.65	5.30

**Table 5:** Pearson correlations between neuropsychological measures and constructed composite scores.

Measure	Composite
*Memory Measures*	Memory composite
CVLT Learning Curve: Trial 5-Trial 1	.487^∗∗^
CVLT Total (Trial 1 through Trial 5)	.804^∗∗^
CVLT Short Delay Free Recall	.853^∗∗^
CVLT Short Delay Cued Recall	.882^∗∗^
CVLT Long Delay Free Recall	.891^∗∗^
CVLT Long Delay Cued Recall	.833^∗∗^
Rey Figure Immediate Recall	.759^∗∗^
Rey Figure Delayed Recall	.767^∗∗^
Rey Figure Recognition	.415^∗^
Digit Span Total Score	.353^∗∗^
Spatial Span Total Score	.343^∗^
*Executive Function Measures*	Executive composite
Symbol Digits Correct—Written	.798^∗∗^
Trail Making Test A (seconds)	-.755^∗∗^
Trail Making Test B (seconds)	-.832^∗∗^
Booklet Category Test (total errors)	-.467^∗^
Wisconsin Card Sort—Total # of Categories	.347
Wisconsin Card Sort—Trials to 1st Category	.012
Wisconsin Card Sort—Failure to Maintain Set	-.108
Wisconsin Card Sort—Learning to Learn (%)	.365^∗^
COWAT total Score	.653^∗∗^
Woodcock-Johnson Test 16—Decision Speed	.667^∗∗^
*Perception and Visual Processing Measures*	Perception composite
Woodcock-Johnson Test 13 Picture Recognition	.881^∗∗^
Woodcock-Johnson Test 3 Spatial Relations	.874^∗∗^
*Conceptual Processing and Reasoning Measures*	Concept/reasoning composite
Woodcock-Johnson Test 15 Analysis/Synthesis	.838^∗∗^
Woodcock-Johnson Test 5 Concept Formation	.813^∗∗^
Woodcock-Johnson Test 1 Verbal Comprehension	.747^∗∗^
*Organization and Attention Measures*	
Rey Figure Score to Copy	-.720^∗∗^
Rey Figure Time to Copy (seconds)	-.709^∗∗^
General Cognitive Functioning	
WASI Verbal	.731^∗∗^
WASI Performance	.831^∗∗^
MMSE	.725^∗∗^

Note: ^∗^*p* < .05; ^∗∗^*p* < .01.

**Table 6:** Pearson correlations between constructed composite scores and categorization measures (older adults only).

Measure	CP Test 1 (pre)	CP T2 (pre)	Probe 1	Probe 2^+^	Probe 3^+^	CP Test 1 (post)^+^	CP Test 2 (post)^+^	Post-pre CP Test 1	Post-pre CP T2	Probe 3-Probe 1
Memory composite	.609^∗∗^	.530^∗∗^	.407^∗^	.697^∗∗^	.089	.323	.277	.235	.273	.461
Executive Functioning composite	.398^∗^	.446^∗^	.365^∗^	.342	.180	.244	.510	.047	.141	.458
Perception/Visual Processing composite	.673^∗∗^	.422^∗^	.227	.824^∗∗^	.120	.740^∗∗^	.503	.064	.468^∗^	.310
Conceptual/Reasoning composite	.595^∗∗^	.437^∗∗^	.614^∗∗^	.721^∗∗^	.290	.541^∗^	.449	.064	.401	.777^∗∗^
Organization/Attention composite	.290	.177	.480^∗∗^	.440	.424	.484	.463	.214	.174	.356
Global Cognitive composite	.609^∗∗^	.517^∗∗^	.343^∗^	.864^∗∗^	.096	.443	.537^∗^	.004	.503^∗^	.596^∗^

Note: Executive Functioning composite does not include the following Wisconsin Card Sort Measures: Total Number of Categories, Trials to 1^st^ Category, and Failure to Maintain Set. Posttests and pre-post difference scores include the experimental older participants only. ^∗^*p* < .05; ^∗∗^*p* < .01. + indicates partial correlations holding the corresponding pretest performance as a covariate.

## Data Availability

The data used to support the findings of this study are available from the corresponding author upon request. Availability is dependent upon compliance with personal data protection laws and ethics board laws and regulations.
